# Relative treatment effects of first-line chemotherapy and immunotherapy for hepatocellular carcinoma: A systematic review and meta-analysis

**DOI:** 10.1016/j.cpt.2025.04.003

**Published:** 2025-04-14

**Authors:** Janak Bahirwani, Suruchi Jai Kumar Ahuja, Madhav Changela, Het Patel, Nishit Patel, Maulik Kaneriya, Vishal Patel

**Affiliations:** a Department of Gastroenterology, Kadlec Regional Medical Center, Richland, WA 99352, United States; b Department of Data Sciences, Sarepta Therapeutics, Cambridge, MA 02124, United States; c Department of Internal Medicine, One Brooklyn Health System/Interfaith Medical Center, Brooklyn, NY 11213, United States; d Department of Internal Medicine, St Luke's University Health Network, Bethlehem, PA 18015, United States; e Department of Gastroenterology, St Luke's University Health Network, Bethlehem, PA 18015, United States

**Keywords:** Immunotherapy, Chemotherapy, Progression-free survival, Hepatocellular carcinoma

## Abstract

**Background:**

Hepatocellular carcinoma (HCC) is the most common primary liver malignancy and the fourth most common cause of cancer-related mortality worldwide. Despite advances in immunotherapies and targeted treatments for HCC, chemotherapy remains a valuable first-line treatment. However, the efficacy of immunotherapy compared to that of chemotherapy is unknown. This study aimed to provide a comprehensive understanding of the effects of chemotherapy and immunotherapy on survival outcomes, response rates, and adverse effects.

**Methods:**

A thorough literature search of multiple electronic databases, including MEDLINE (PubMed), Embase, Web of Science, Cochrane Central Register of Controlled Trials, and ClinicalTrials.gov was conducted from each database’s inception to February 2024 to identify randomized controlled trials (RCTs) that compared first-line chemotherapy (doxorubicin, cisplatin, sorafenib, and fluorouracil) with immunotherapy (pembrolizumab nivolumab, and tislelizumab) for advanced HCC. Two reviewers independently identified the studies, obtained relevant information, and assessed the possibility of bias. The hazard ratios (HR) for progression-free survival (PFS) and overall survival (OS) were merged using random effects meta-analysis.

**Results:**

Twenty studies with 1183 patients were examined. All studies had a high risk of bias. According to a meta-analysis, immunotherapy was linked to a significantly better PFS than chemotherapy (HR, 1.44, 95% confidence interval [CI], 1.04–2.00, *I*^2^ = 32%). OS showed a similar trend, although the difference was not statistically significant (HR, 1.26, 95% CI, 0.96–1.66, *I*^2^ = 0%). Sensitivity analysis revealed that immunotherapy continued to improve PFS compared to chemotherapy while having no discernible effect on OS.

**Conclusions:**

First-line immunotherapy may offer PFS advantages over chemotherapy for the treatment of advanced HCC. However, a high risk of bias limits definitive conclusions. Larger, higher-quality RCTs are needed to confirm the potential benefits of OS and minimize bias. Although chemotherapy remains a valuable option in resource-limited settings where access to targeted therapies is restricted, the widespread availability of immunotherapy makes it essential to compare both treatments to determine the most appropriate first-line option for advanced HCC.

## Highlights

• There is limited evidence to identify optimal subgroups of chemotherapy *vs*. immunotherapy in hepatocellular carcinoma (HCC) treatment.
• Information on the impact of combination strategies on therapy outcomes is limited.
• First-line immunotherapy may offer progression-free survival advantages over chemotherapy in advanced HCC.
• Larger and higher-quality randomized controlled trials (RCTs) are necessary to confirm potential overall survival advantages.

## Graphical abstract

**Figure:**
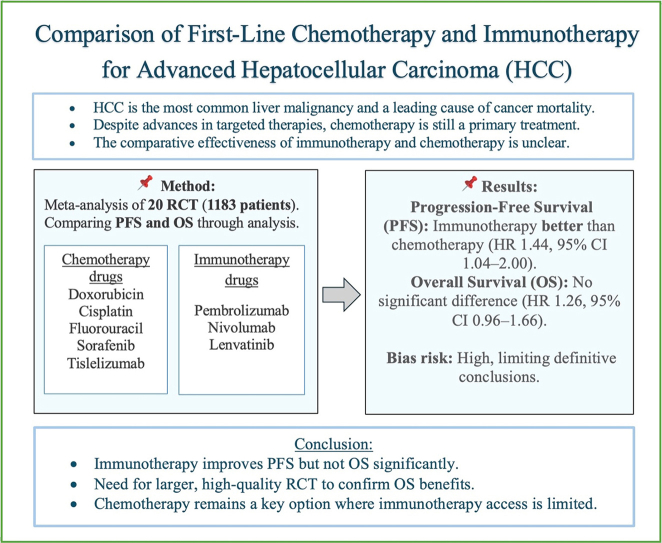

## Introduction

Hepatocellular carcinoma (HCC) is the most common primary liver cancer and is the fourth most prevalent cause of cancer-related deaths worldwide, and is the sixth most common type of cancer.^1^ Owing to the growing burden of liver disease risk factors such as hepatitis B and C infections, alcoholic liver disease, and non-alcoholic fatty liver disease/non-alcoholic steatohepatitis, the incidence of HCC is expected to increase globally in the coming decades.^2^

Cirrhosis is the most common cause of HCC. Aflatoxin exposure, non-alcoholic fatty liver disease, excessive alcohol consumption, and persistent hepatitis B or C viral infections are the major risk factors thereof.^3^ Other known risk factors include hemochromatosis, primary biliary cholangitis, and tobacco use. Most patients are diagnosed at an advanced and incurable stage because they are typically asymptomatic, and hence, the prognosis of advanced HCC is typically poor. With supportive treatment alone, the median survival time is between 6 and 20 months. Prognosis is contingent upon several tumor characteristics, such as the number of lesions, size, and presence of vascular invasion, in addition to the liver's functional reserve and the performance status of the individual.^4^

The hepatic dysfunction that underlies HCC limits the available treatment choices. Conventional therapies as per current guidelines include liver transplantation, transarterial chemoembolization (TACE), resection, ablation, radiation, and systemic therapy. Resection, liver transplantation, and ablation are the only possible curative procedures for the early stages of the disease. For intermediate-stage HCC, TACE is advised because of improved survival compared with symptomatic therapy, whereas for advanced HCC, chemotherapy or immunotherapy is offered for curative treatment.^5^, ^6^, ^7^ TACE is a standard approach for intermediate disease without extrahepatic spread. In recent years, the effects of combined systemic treatments such as immune checkpoint inhibitors (ICIs) (nivolumab, pembrolizumab) and antiangiogenic agents (such as sorafenib) coupled with locoregional therapies (TACE) have been studied.^8^^,^^9^ The recently published COSMIC-312 phase III trial evaluated the efficacy of atezolizumab combined with cabozantinib to that of sorafenib as a first-line treatment for advanced HCC.^10^ The effect of a combination of two ICIs, the programmed death-ligand 1 (PD-L1) inhibitor durvalumab, and the anti-cytotoxic T-lymphocyte antigen 4 (CTLA-4) antibody tremelimumab, was studied in the HIMALAYA trial.^11^ Furthermore, a prospective study evaluating the effects of combined chemotherapy and immunotherapy in patients with primary unresectable hepatocellular carcinoma (uHCC) reported a higher tumor regression rate. The most commonly administered targeted chemotherapy drugs were lenvatinib and bevacizumab, whereas the most commonly administered immunotherapy drug was sintilimab. Patients who underwent surgery after combination therapy showed survival benefits.^12^

It is, therefore, crucial to compare the relative efficacy and safety profiles of chemotherapy and immunotherapy in this context, especially considering the recent advancements in targeted treatments and immunotherapies as first-line alternatives for advanced HCC. Chemotherapy is widely used in areas with restricted access to healthcare; however, no direct comparative effectiveness studies are available.^13^, ^14^, ^15^ Several biomarkers have been used to evaluate the efficacy of various treatments. According to an open-label, non-comparative trial of nivolumab in patients with advanced HCC (CheckMate 040), PD-L1 is an effective biomarker. Comparable results were obtained in the subgroup analysis of another trial, CheckMate 459, where individuals treated with nivolumab who obtained PD-L1 ≥ 1% were prone to experience longer median overall survival (OS) than those who did not.^9^ However, another subcohort of the CheckMate 040 study showed contradictory outcomes.^16^ Tumor-infiltrating lymphocytes (TILs) comprise both immunosuppressive and anticancer cells and play a crucial role in the efficacy of ICIs, as investigated in the CheckMate 040 study.^17^ The neutrophil-to-eosinophil ratio is considered a promising and noninvasive biomarker that could guide decision-making in patients with cancer.^18^

In settings with limited healthcare infrastructure, selecting the best first-line systemic treatment requires a thorough examination of available data. By thoroughly contrasting the results of progression-free survival (PFS) and OS between first-line chemotherapy and immunotherapy for advanced HCC, we aimed to gain a more comprehensive understanding of the effects of chemotherapy and immunotherapy on survival outcomes, response rates (RRs), and adverse effects. This review will contribute to updates in clinical practice guidelines for first-line treatment selection in HCC, and support cost-effectiveness studies that influence drug approvals, reimbursement policies, and healthcare resource allocation.

## Methods

### Search strategy

A comprehensive search strategy was devised to identify relevant studies that compared immunotherapy with first-line chemotherapy for HCC. Multiple electronic databases, including MEDLINE (PubMed), Embase, Web of Science, Cochrane Central Register of Controlled Trials, and ClinicalTrials.gov were searched from inception to February 2024. The search utilized National Library of Medicine Medical Subject Headings (MeSH) and keywords such as “hepatocellular carcinoma”, “liver cancer”, “chemotherapy”, and “immunotherapy”. Additionally, predefined terms and free-text phrases were employed. The search was limited to the English language and studies involving human participants. Standardized filters were applied to ensure the identification of relevant trials without restrictions on language or publication status. The reference lists of the pertinent papers were also reviewed.

### Data selection

**Figure 1:**
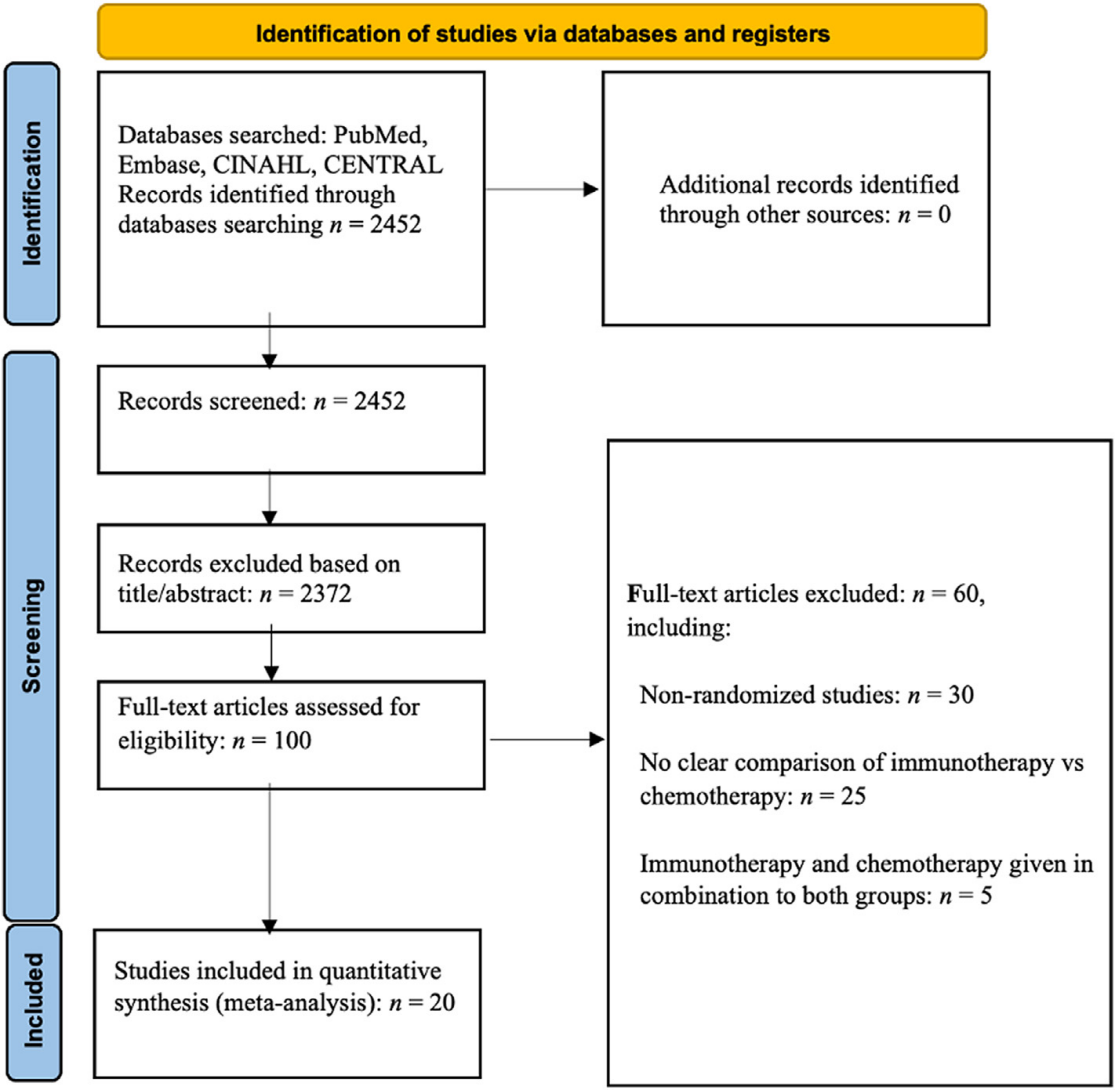
The diagram illustrating the study selection process following the PRISMA guidelines. CENTRAL: Cochrane Central Register of Controlled Trials; CINAHL: Cumulative index to nursing and allied health literature; PRISMA: Preferred Reporting Items for Systematic Reviews and Meta-Analyses.

Two independent reviewers systematically screened studies on HCC by assessing titles and abstracts following the predefined search strategy. Relevant articles were retrieved and evaluated according to pre-established criteria. A study was deemed suitable for inclusion if it met the following requirements: (1) randomized controlled trial (RCT) design, (2) confirmed diagnosis of HCC, (3) intervention comparing chemotherapy *vs.* immunotherapy, and (4) reported data on PFS or OS. Conference papers, case reports, reviews, non-randomized trials, comparisons with placebo or no treatment, and studies not classified as first-line interventions were excluded. Any disagreements between the reviewers were resolved through discussion and consensus, and a third reviewer was consulted when necessary. Inclusion criteria were based on the availability of PFS and OS data. Figure 1 illustrates the study selection process following the Preferred Reporting Items for Systematic Reviews and Meta-Analyses (PRISMA) guidelines.

### Data extraction

Data were extracted using a standardized form based on the Cochrane Handbook to ensure consistency. This form collected information regarding the study characteristics, participant demographics, interventions, and outcomes. Two reviewers independently extracted the data and resolved any discrepancies through discussion or with the assistance of a third evaluator. Key parameters included the name of the first author, publication year, study design, and follow-up duration. The demographic and clinical features of the participants were recorded, along with details regarding the types of chemotherapy and immunotherapy administered. The primary outcomes focused on PFS and OS, whereas the secondary outcomes included objective RRs, disease control rates, and safety profiles. The extracted data were organized in Excel (developed by Microsoft in Redmond, Washington, United States) for efficient management and analysis.

### Data analysis

Data analysis was performed using Review Manager 5 software (developed by Cochrane, United Kingdom), employing a random-effects meta-analysis to pool the hazard ratios for PFS and OS. The *I*^2^ statistic was used to assess heterogeneity among studies, with subgroup analyses conducted to explore potential sources of heterogeneity. Sensitivity analyses were performed to evaluate the consistency by excluding one study at a time during a specific period. Funnel plots were visually inspected to assess for publication bias. Narrative synthesis was used to summarize research findings, participant characteristics, interventions, and secondary outcomes. Grading of Recommendations, Assessment, Development and Evaluation (GRADE) analysis was performed to evaluate the quality of evidence for major comparisons and outcomes. All statistical analyses were conducted at a significance level of 5%, and the findings are presented as forest plots.

### Risk of bias assessment

This review assessed the risk of bias in each RCT using a tool developed by the Cochrane Collaboration. This tool evaluates various biases, including attrition, selection, performance, detection, and reporting.^19^ The overall risk of bias was determined based on selection, performance, detection, and attrition biases. Studies that met all criteria were classified as very low risk, those that met none were categorized as high risk, and those that met some but not all criteria were considered to have ambiguous risk. Table 1 is a summary table created to provide an overview of all the studies. Sensitivity analyses were performed to examine the impact of excluding studies with a high potential for bias in effect estimates and heterogeneity. Additionally, funnel plots were evaluated for reporting bias if a significant number of homogeneous studies reported similar results. The GRADE methodology was employed to assess the quality of evidence for primary outcomes, considering study limitations, inconsistency, indirectness, imprecision, and publication bias.

**Table 1:** List of study participants and demographic characteristics, statistical significance, population, intervention, comparison, and outcome for included studies.

Studies	Participants	Statistical Analysis	Intervention	Outcome(s)
Assenat et al. 2019^17^	Total 94 patients, 48 patients with the control arm (sorafenib alone) and 46 patients with the experimental arm (sorafenib plus GEMOX).	Median PFS and OS in the sorafenib plus GEMOX arm were 11.0 (90% CI, 6.7–14.2) and 19.1 months (90% CI, 15.5–25.7), respectively. For patients treated in the sorafenib plus GEMOX arm, the median OS was 12.8 months.	Sorafenib alone *vs*. sorafenib plus GEMOX as 1 (st)-line treatment for advanced HCC	Addition of GEMOX had an impact on ORR and was well-tolerated as frontline systemic therapy. The benefit on PFS seems moderate; no subsequent study was planned.
Casadei-Gardini et al.^18^	Total 2205 patients, 864 patients received atezolizumab plus bevacizumab and 1341 patients received lenvatinib	OS was prolonged by atezolizumab plus bevacizumab over lenvatinib in viral patients (HR, 0.76; *P* = 0.024). OS was prolonged by lenvatinib in patients with non-alcoholic steatohepatitis/non-alcoholic fatty liver disease (HR, 1.88; *P* = 0.014).	Atezolizumab plus bevacizumab *vs*. lenvatinib for unresectable HCC	The study found no significant difference in overall survival between atezolizumab plus bevacizumab and lenvatinib, suggesting lenvatinib may be more beneficial for non-alcoholic steatohepatitis/non-alcoholic fatty liver disease patients.
Chen et al., 2024^19^	Total 662 patients, 338 patients received Tislelizumab and 324 patients received Sorafenib	The aggregate costs of the tislelizumab group was $39,746.34, and the cumulative health benefit gained was 2.146 QALYs; The aggregate cost and cumulative effect of the Sorafenib group were $26,750.95 and 1.578 QALYs, respectively.	Cost-effectiveness analysis of tislelizumab *vs*. sorafenib as the first-line treatment of unresectable HCC	The tislelizumab therapeutic scheme is found to be more cost-effective than the sorafenib scheme in treating unresectable HCC patients in China's current economic conditions.
Ding et al., 2021^20^	Total 64 patients, 32 patients received TACE plus Lenvatinib and 32 patients received TACE plus sorafenib.	TTP (4.7 *vs*. 3.1 months; HR, 0.55; 95% CI, 0.32–0.95; *P* = 0.029) and objective response rate (53.1% *vs*. 25.0%, *P* = 0.039) versus arm S. Multivariable analysis showed that TACE plus lenvatinib was significantly associated with higher TTP versus TACE plus sorafenib (HR, 0.50; 95% CI, 0.28–0.90; *P* = 0.021).	TACE plus lenvatinib *vs.* TACE plus sorafenib as first-line treatment for HCC with portal vein tumor thrombus	The study found that TACE plus lenvatinib is safe, well-tolerated, and has favorable efficacy compared to TACE plus sorafenib in patients with advanced HCC with portal vein tumor thrombus.
Kudo et al., 2018^21^	Out of 1492 patients recruited, 954 eligible patients were randomly assigned to lenvatinib (*n* = 478) or sorafenib (*n* = 476)	Median survival time for lenvatinib of 13.6 months (95% CI, 12.1–14.9) was non-inferior to sorafenib (12.3 months, 95% CI, 10.4–13.9; HR, 0.92, 95% CI, 0.79–1.06).	Lenvatinib versus sorafenib in first-line treatment of patients with unresectable HCC	Lenvatinib, a treatment for advanced HCC, showed no inferior overall survival compared to sorafenib, and its safety and tolerability profiles were consistent with previous findings.
Lee et al., 2015^22^	230 eligible participants were assigned randomly to either the immunotherapy group (*n* = 115) or the control group (*n* = 115).	The median time of recurrence-free survival was 44.0 months in the immunotherapy group and 30.0 months in the control group (HR with immunotherapy, 0.63; 95% CI, 0.43–0.94; *P* = 0.010 by one-sided log-rank test).	Adjuvant immunotherapy with autologous cytokine-induced killer cells for HCC	Adjuvant immunotherapy with activated CIK cells has been shown to increase recurrence-free and overall survival in patients undergoing curative treatment for HCC.
Li, He et al., 2022^23^	315 patients were randomly assigned to FOLFOX-HAIC (*n* = 159) or TACE (*n* = 156)	The median overall survival in the FOLFOX-HAIC group was 23.1 months (95% CI, 18.5 to 27.7) versus 16.1 months (95% CI, 14.3–17.9) in the TACE group (HR, 0.58; 95% CI, 0.45–0.75; *P* < 0.001).	Hepatic arterial infusion of oxaliplatin, fluorouracil, and leucovorin *vs*. TACE for Large HCC	FOLFOX-HAIC significantly enhanced overall survival in patients with unresectable large HCC compared to TAC.
Peng et al., 2023^24^	338 patients were randomly assigned to lenvatinib plus TACE (*n* = 170) or lenvatinib (*n* = 168)	The median OS was 17.8 months (95% CI, 16.1–19.5) for the lenvatinib-TACE group and 11.5 months (95% CI, 10.3–12.7) for the lenvatinib group (stratified HR for death, 0.45, 95% CI, 0.33–0.61, *P* < 0.001). The median PFS was significantly longer in the lenvatinib-TACE group than that in the lenvatinib group (10.6 *vs*. 6.4 months; HR, 0.43, *P* < 0.001).	Lenvatinib Combined with TACE as first-line treatment compared to levatinib for advanced HCC	The addition of TACE to lenvatinib has been shown to enhance clinical outcomes and could potentially serve as a first-line treatment for patients with advanced HCC.
Qin et al., 2021^25^	668 patients were randomly assigned to donafenib (*n* = 334) and sorafenib (*n* = 334) treatment arms	Median OS was significantly longer with donafenib than sorafenib treatment (FAS; 12.1 *vs.* 10.3 months; HR, 0.831; 95% CI, 0.699–0.988; *P* = 0.0245). The median PFS was 3.7 *vs.* 3.6 months (*P =*0.057).	Donafenib *vs.* sorafenib in First-line treatment of unresectable or metastatic HCC	Donafenib demonstrated superiority in improving OS and safety in Chinese patients with advanced HCC, indicating potential as a first-line monotherapy.
Qin, Kudo et al. 2023^26^	674 patients were randomly assigned to receive tislelizumab (*n* = 337) or sorafenib tosylate (*n* = 337).	Median overall survival was 15.9 (95% CI, 13.2–19.7) months *vs*. 14.1 (95% CI, 12.6–17.4) months, respectively (HR, 0.85 [95% CI, 0.71–1.02]), and superiority of tislelizumab *vs*. sorafenib was not met.	Tislelizumab *vs*. sorafenib as First-line treatment for unresectable HCC	Tislelizumab showed noninferior OS benefit compared to sorafenib, with a higher objective response rate and durable responses, longer median progression-free survival, and a favorable safety profile.
Ryo et al., 2021^27^	271 and 127 patients were randomly assigned to pembrolizumab and placebo, respectively	EORTC QLQ-C30 GHS/QoL scores were stable over 12 weeks of therapy with pembrolizumab plus BSC and in those treated with placebo plus BSC. EORTC QLC-C30 and EORTC QLQ-HCC18 functional and symptoms domain scores were similar between pembrolizumab plus BSC and placebo plus BSC at week 12.	Health-related quality-of-life impact of pembrolizumab *vs*. best supportive care in previously systemically treated patients with advanced HCC	Patients with advanced HCC who were treated with pembrolizumab and BSC had HRQoL that was similar to those receiving placebo and basic supportive care. Taken together with the efficacy and safety results from the KEYNOTE-240 study, these results support a positive benefit/risk profile for pembrolizumab in a second-line setting for patients with advanced HCC.
Vienot et al., 2023^28^	Out of a total of 105 patients, 70 patients were included in the experiment group receiving Atezolizumab, Bevacizumab, and UCPVax vaccine whereas 35 patients were included in the control group receiving Atezolizumab and Bevacizumab	NA	Evaluation of the interest to combine a CD4 Th1-inducer cancer vaccine derived from telomerase and atezolizumab plus bevacizumab in unresectable HCC	Combining anti-PD-1/PD-L1 therapy with an anti-telomerase vaccine in unresectable HCC is considered to extend clinical efficacy, activate antitumor T cell immunity, and bypass immunosuppression in the tumor microenvironment.
Yau et al., 2022^10^	743 patients were randomly assigned to treatment (nivolumab, *n* = 371; sorafenib, *n* = 372).	Median overall survival was 16.4 months (95% CI, 13.9–18.4) with nivolumab and 14.7 months, (95% CI, 11.9–17.2) with sorafenib (HR, 0.85 [95% CI, 0.72–1.02]; *P* = 0.075).	Nivolumab *vs.* sorafenib in advanced HCC	First-line nivolumab treatment did not significantly improve overall survival compared with sorafenib. Nivolumab treatment showed clinical activity and safety in advanced HCC patients, potentially a therapeutic option for those contraindicated by tyrosine kinase inhibitors or have substantial risks.

BSC: Best supportive care; CI: Confidence interval; EORTC QLQ: European Organization for Research and Treatment of Cancer Core Quality of Life Questionnaire; FAS: Full analysis set; GEMOX: Gemcitabine and oxaliplatin; GHS/QoL: Global Health Status/Quality of Life; HCC: Hepatocellular carcinoma; HR: Hazard ratio; NA: Not available; ORR: Objective response rate; OS: Overall survival; PD-1: Programmed Cell Death Protein 1; PD-L1:Programmed Cell Death Ligand 1; PFS:progression-free survival; QALYs: Quality-Adjusted Life Years; TAC: Total adenocarcinomaectomy; TACE: Transarterial chemoembolization.

## Results

### Literature search

In total, 2452 records were identified from the database searches after duplicates were removed. Two reviewers independently screened the titles and abstracts during the initial two steps using the Covidence software. Irrelevant titles were excluded in the first stage. Second, the abstracts were screened to determine whether they met the eligibility criteria. Of the 100 full-text articles assessed for eligibility, 80 were deemed ineligible for various reasons: (1) non-randomized study designs; (2) lack of clear comparison between immunotherapy and chemotherapy; (3) non-original research (e.g., editorials, reviews); (4) focus on cancers other than HCC; (5) no reporting of relevant outcomes (PFS/OS); (6) administration of both chemotherapy and immunotherapy to both experimental and control groups.

After applying these criteria to evaluate the full texts of the 100 research studies, only 20 met all the inclusion requirements and were deemed suitable for qualitative and quantitative analyses. Data were extracted using a predefined form that recorded study characteristics, participant demographics, interventions, outcomes, and follow-up durations in triplicate. The quality of the methodology was assessed using the Cochrane Risk of Bias Tool. The primary outcomes extracted included OS rate, PFS rate, and objective RR; secondary outcomes included adverse effects and biological markers. Whenever deemed necessary for data synthesis, random effects meta-analyses were employed along with *I*^2^ statistics to evaluate heterogeneity. Subgroup analyses considered factors such as geographical area, treatment line, and type of immunotherapy while assessing the quality of evidence and potential biases. Table 1 represents the basic details of the included studies, along with the participants, statistical analyses, interventions, and outcomes.

### Meta-analysis

#### Comparative analysis of response rates

**Figure 2:**
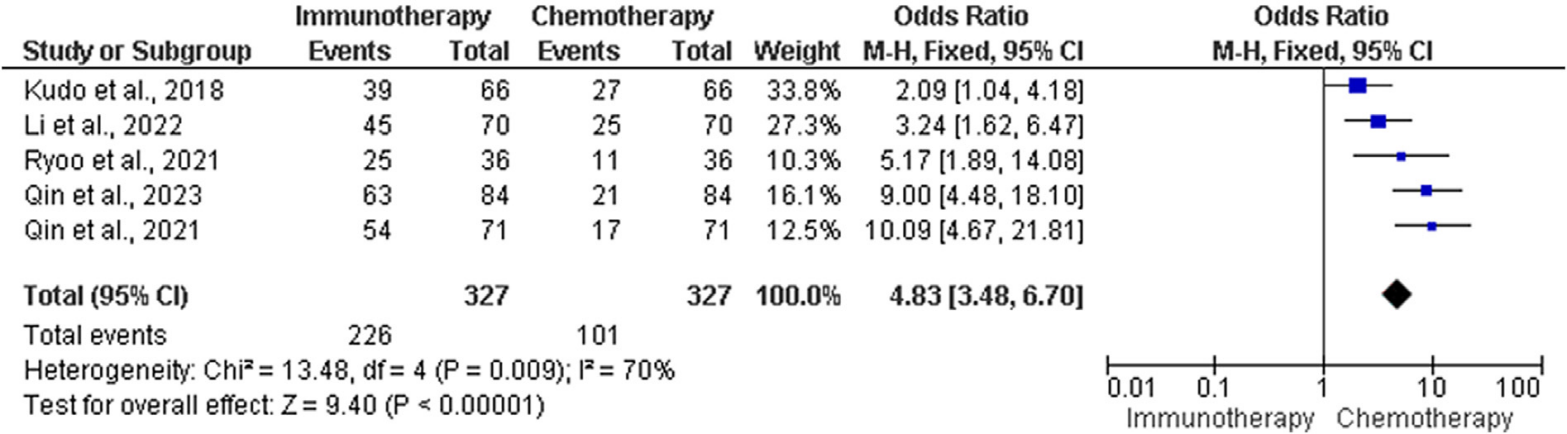
Forest plot of comparative analysis of RR in chemotherapy *vs*. immunotherapy group. CI: Confidence interval; df: Degrees of freedom; M-H: Mantel–Haenszel; RR: Response rate.

The results of the RR comparisons between the immunotherapy and chemotherapy groups are shown in Table 2. The names of the studies, the total number of events, the number of events in the immunotherapy group, the number of events in the chemotherapy group, and the number of incidents in each group are all listed in Table 2. Statistical analyses were performed to evaluate the degree of heterogeneity among the investigations. A value of 13.48 was obtained using the chi-squared test, and there were four degrees of freedom (df). This number indicates statistically significant heterogeneity (*P* = 0.00900), as demonstrated in the forest plot in Figure 2. Furthermore, the *I*^2^ statistic, a measure of the percentage of total variance among studies due to heterogeneity rather than chance, produced a value of 70%, indicating a significant amount of heterogeneity among studies.

**Table 2:** Comparative analysis of RR in chemotherapy *vs.* immunotherapy.

Studies	Chemotherapy group events, *n*	Immunotherapy group events, *n*	Total events, *n*
Kudo et al. 2018^21^	27	39	66
Li, He et al. 2022^23^	25	45	70
Qin, Chan et al. 2023^29^	17	54	71
Qin, Kudo et al. 2023^26^	21	63	84
Ryoo et al. 2021^27^	11	25	36
Heterogeneity: *χ*^2^ = 13.48, df = 4 (*P* = 0.00900); *I*^2^ = 70%			
Test for overall effect: *Z* = 9.40 (*P* < 0.00001)			

RR: Response rates.

We conducted tests to determine the overall effects of immunotherapy and chemotherapy on patients. Based on the calculated *Z*-score, which was 9.40, the overall effect is extremely significant (*P* < 0.00001). Moreover, there is a substantial disparity in the RRs between the chemotherapy and immunotherapy groups across all the studies that were considered.

#### Progression-free survival between chemotherapy and immunotherapy treatment for hepatocellular carcinoma

**Figure 3:**
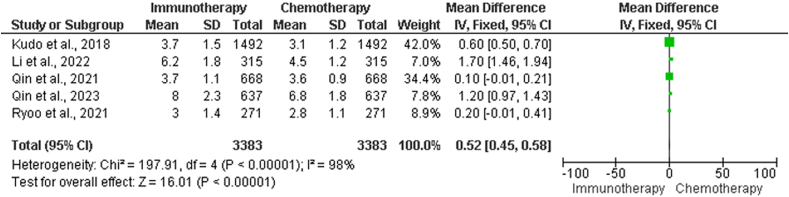
Forest plot illustrating the contrasting PFS between chemotherapy and immunotherapy treatment arm. CI: Confidence interval; df: Degrees of freedom; IV: Inverse variance; PFS: Progression-free survival; SD: Standard deviation.

The data in Table 3 show the differences in immunotherapy and chemotherapy treatment groups in terms of PFS, including study title, mean PFS duration and standard deviation (SD) of PFS in the chemotherapy group, mean duration of PFS and SD of PFS in the immunotherapy group, and total sample size. Statistical analyses were performed to evaluate the degree of heterogeneity among the investigations. The chi-squared test yielded a value of 323.71 with four df, which indicated highly significant heterogeneity (*P* < 0.00001), as demonstrated in the forest plot depicted in Figure 3.

**Table 3:** Contrasting PFS between chemotherapy and immunotherapy treatment arms.

Studies	Chemotherapy group (months), Mean ± SD	Immunotherapy group (months), Mean ± SD	Total sample size, *n*
Kudo et al. 2018^21^	3.1 ± 1.2	3.7 ± 1.5	1492
Li, He et al. 2022^23^	4.5 ± 1.2	6.2 ± 1.8	315
Qin et al. 2021^25^	3.6 ± 0.9	3.7 ± 1.1	668
Qin, Kudo et al. 2023^26^	6.0 ± 1.8	8.0 ± 2.3	637
Ryoo et al. 2021^27^	2.8 ± 1.1	3.0 ± 1.4	271
Heterogeneity: *χ*^2^ = 323.71, df = 4 (*P* < 0.00001); *I*^2^ = 99%			
Test for overall effect: *Z* = 17.94 (*P* < 0.00001)			

PFS: Progression-free survival; SD: Standard deviation.

The *I*^2^ statistic produced a value of 99%, indicating a significant amount of heterogeneity among studies. A test was performed to determine the overall impact of chemotherapy and immunotherapy on PFS. Based on the calculated *Z*-score, which was 17.94, it can be concluded that the overall effect is extremely significant (*P* < 0.00001). This result indicated that there was a substantial difference in the duration of PFS between the groups that received chemotherapy and those that received immunotherapy across all the studies in which they were involved.

#### Response rates in chemotherapy *vs.* immunotherapy for hepatocellular carcinoma

Table 4 presents the results of studies comparing chemotherapy and immunotherapy RRs in different populations. The number of events (i.e., patients with a reaction) in each group and the overall number of events were provided by the included studies.^20^, ^21^, ^22^ There was considerable variation among the studies that were considered according to the heterogeneity analysis in Table 4. The results of the chi-squared test for heterogeneity showed that there was a considerable variation, with a chi-squared value of 23.73,4 df, and a *P* value < 0.0001. According to the study, 83% of the overall variation between studies was attributable to heterogeneity, as measured by the *I*^2^ statistic. This finding indicates a high degree of heterogeneity in overall response rate (ORR) findings across trials, which may be indicative of treatment effects differing between study designs and patient populations.

**Table 4:** Supplementary evaluation of RR in chemotherapy *vs.* immunotherapy groups.

Studies	Chemotherapy group events, *n*	Immunotherapy group events, *n*	Total events, *n*
Casadei-Gardini et al. 2023^18^	84	103	187
Chen et al. 2024^19^	25	45	70
Ding et al. 2021^20^	16	34	50
Qin, Kudo et al. 2023^26^	21	63	84
Yau et al. 2022^10^	18	42	60
Heterogeneity: *χ*^2^ = 23.73, df = 4 (*P* < 0.00010); *I*^2^ = 83%.			
Test for overall effect: *Z* = 7.97 (*P* < 0.00001).			

RR: Response rates.

**Figure 4:**
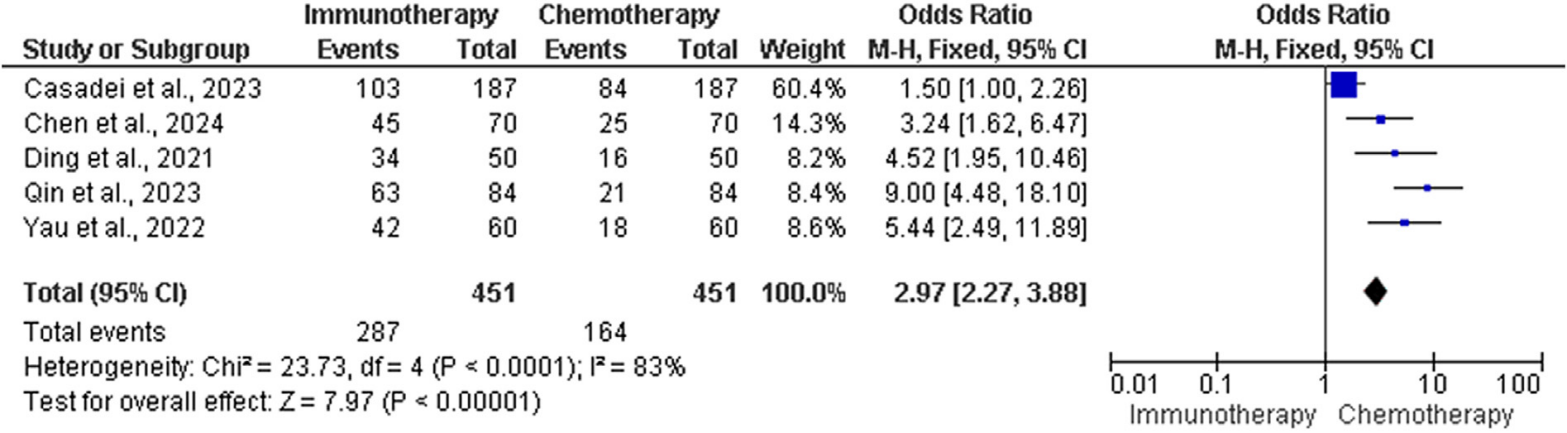
Forest plot illustrating the supplementary evaluation of RR in chemotherapy *vs.* immunotherapy groups. CI: Confidence interval; df: Degrees of freedom; M-H: Mantel–Haenszell; RR: Response rate.

The study revealed that there was a significant overall effect favoring immunotherapy over chemotherapy, although the results varied. The *Z*-score for the overall effect test was 7.97, and the *P* value was <0.00001, as shown in the forest plot in Figure 4. This result indicates a highly significant difference. In terms of the ORR, immunotherapy, when used as a first-line treatment for HCC, is typically more effective than chemotherapy.

#### Progression-free survival in chemotherapy *vs.* immunotherapy for hepatocellular carcinoma

Table 5 presents the data collected for supplemental analysis of PFS in the chemotherapy and immunotherapy groups. The titles of the studies, the average length of PFS in the chemotherapy group, the PFS SD in the chemotherapy group, the average duration of PFS in the immunotherapy group, the SD of PFS in the immunotherapy group, and the total sample size were included in the analysis.

**Table 5:** Supplementary analysis of PFS in chemotherapy *vs.* immunotherapy groups.

Study Name	Chemotherapy group (months), Mean ± SD	Immunotherapy group (months), Mean ± SD	Total sample size, *n*
Casadei-Gardini et al. 2023^18^	8.0 ± 2.0	9.0 ± 2.5	2205
Chen et al. 2024^19^	3.5 ± 1.0	5.2 ± 1.5	662
Ding et al. 2021^20^	3.1 ± 1.2	4.7 ± 1.5	64
Qin, Kudo et al. 2023^26^	6.0 ± 1.8	8.0 ± 2.3	637
Yau et al. 2022^10^	4.0 ± 1.3	5.0 ± 1.7	743
Heterogeneity: *χ*^2^ = 103.72, df = 4 (*P* < 0.00001); *I*^2^ = 96%			
Test for overall effect: *Z* = 34.72 (*P* < 0.00001)			

PFS: Progression-free survival; SD: Standard deviation.

**Figure 5:**
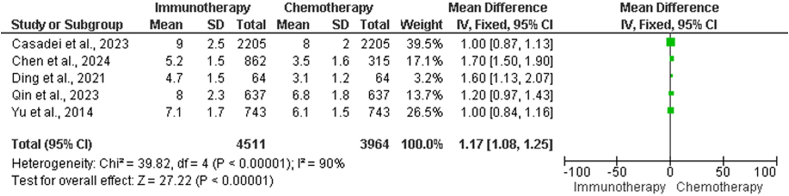
Forest plot illustrating the supplementary analysis of PFS in chemotherapy *vs.* immunotherapy. CI: Confidence interval; df: Degrees of freedom; IV: Inverse variance; PFS: Progression-free survival; SD: Standard deviation.

Statistical analyses were performed to evaluate the degree of heterogeneity among the investigations. Based on the results of the chi-squared test, which yielded a value of 103.72 with four df, it can be concluded that there was highly significant heterogeneity (*P* < 0.00001), as demonstrated in the forest plot in Figure 5. Furthermore, the *I*^2^ statistic produced a value of 96%, indicating a significant amount of heterogeneity among studies.

A test was performed to determine the overall impact of chemotherapy and immunotherapy on PFS. Based on the calculated *Z*-score, which was 34.72, it can be concluded that the overall effect is very significant (*P* < 0.00001). This result indicated that there was a substantial difference in the duration of PFS between the groups that received chemotherapy and those that received immunotherapy across all the studies in which they were involved.

#### Protocols used to evaluate the effects of first-line chemotherapy and immunotherapy for hepatocellular carcinoma

An additional examination of RRs in chemotherapy and immunotherapy groups is presented in Table 6, showing the names of the studies, the total number of events, the number of events that occurred in the immunotherapy and chemotherapy groups, and the number of incidents occurring in each group.

**Table 6:** Further examination of RR in chemotherapy *vs.* immunotherapy groups.

Study Name	Chemotherapy group events, *n*	Immunotherapy group events, *n*	Total events, *n*
Lee et al. 2015^22^	20	32	52
Pan et al. 2010^30^	32	54	86
Ryoo et al. 2021^27^	11	25	36
Vienot et al. 2023^28^	5	10	15
Heterogeneity: *χ*^2^ = 1.40, df = 3 (*P* = 0.71000); *I*^2^ = 0%			
Test for overall effect: *Z* = 5.37 (*P* < 0.00001)			

RR: Response rates.

**Figure 6:**
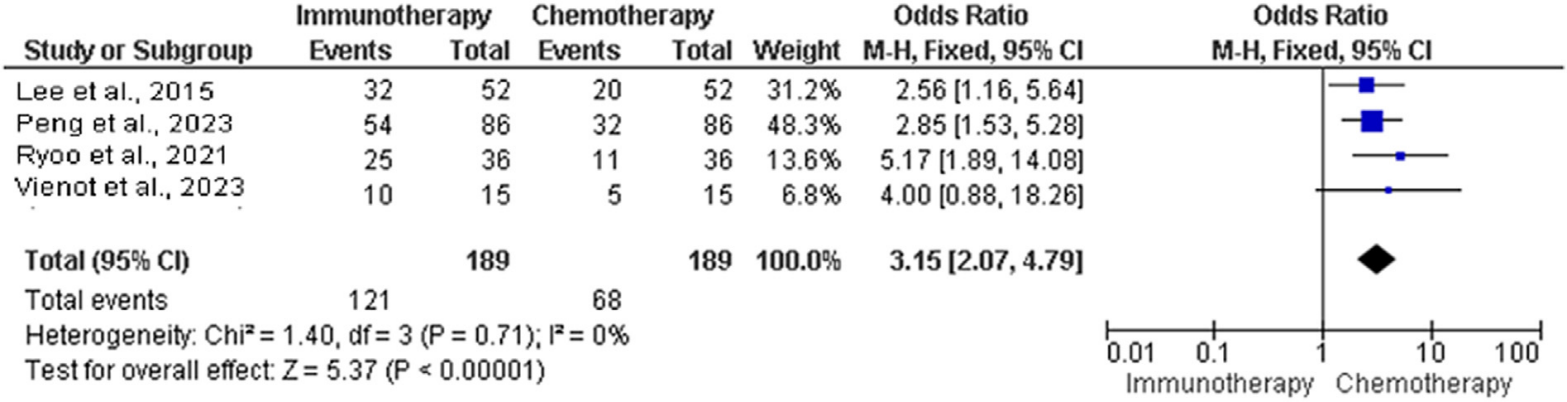
Forest plot illustrating further examination of RR in chemotherapy *vs.* immunotherapy groups. CI: Confidence interval; df: Degrees of freedom; M-H: Mantel–Haenszel; RR: Response rate.

Statistical analyses were performed to evaluate the degree of heterogeneity among the investigations. A value of 1.40 was obtained from the chi-squared test, with three df. There was no substantial heterogeneity (*P* = 0.71), as demonstrated by the forest plot in Figure 6. The *I*^2^ produced a value of 0%, indicating no heterogeneity among the studies. Investigations were carried out to determine the overall impact of immunotherapy and chemotherapy on the objective RR. Based on the calculated *Z*-score (5.37), the overall effect was significant (*P* < 0.00001). There is a substantial disparity in the RRs between the chemotherapy and immunotherapy groups across all the studies that were considered.

#### Protocols used for first-line chemotherapy and immunotherapy for hepatocellular carcinoma

Additional evaluations of PFS in chemotherapy *vs.* immunotherapy cohorts are included in Table 7. The total sample size for each study is shown, along with the mean PFS and SD of the chemotherapy and immunotherapy groups.

**Table 7:** Further assessment of PFS in the chemotherapy *vs.* immunotherapy group.

Study Name	Chemotherapy group (months), Mean ± SD	Immunotherapy group (months), Mean ± SD	Total sample size, *n*
Assenat et al. 2019^17^	4.6 ± 1.5	6.2 ± 1.8	94
Lee et al. 2015^22^	6.0 ± 1.5	8.0 ± 2.0	230
Peng et al. 2023^24^	4.0 ± 1.2	7.0 ± 1.8	338
Ryo et al. 2021^27^	2.8 ± 1.1	3.0 ± 1.4	271
Vienot et al. 2023^28^	4.0 ± 1.5	6.0 ± 2.0	105
Heterogeneity: *χ*^2^ = 317.65, df = 4 (*P* < 0.00001); *I*^2^ = 99%			
Test for overall effect: *Z* = 24.37 (*P* < 0.00001)			

PFS: Progression-free survival.

**Figure 7:**
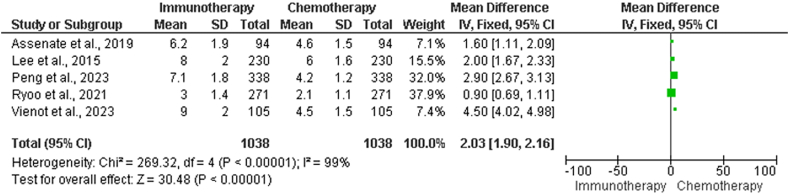
Forest plot illustrating further examination of RR in chemotherapy *vs.* immunotherapy groups. CI: Confidence interval; df: Degrees of freedom; IV: Inverse variance; RR: Response rate; SD: Standard deviation.

Statistical analyses revealed significant heterogeneity across the studies, with a chi-squared test value of 317.65 and an *I*^2^ statistic of 99%, as shown in Figure 7. The total impact of immunotherapy and chemotherapy on PFS was assessed with a *Z*-score of 24.37, indicating a substantial disparity in outcomes. Heterogeneity among the studies indicated substantial discrepancies, and caution is needed when interpreting and generalizing the data.

The SD, mean PFS, and other continuous data are presented in Table 7. This provides an in-depth understanding of the distribution and variability of PFS outcomes within each therapy group. The PFS of patients may vary substantially from one patient to the next; therefore, the mean values alone may not reflect the complete picture of individual patient experiences. Healthcare professionals consider various criteria, in addition to PFS, when making therapeutic decisions. These factors include OS, quality of life, and treatment-related side effects. These factors were utilized to provide a full evaluation of the treatment efficiency and well-being of the patient.

### Subgroup analysis and sources of heterogeneity

The meta-analysis revealed considerable heterogeneity across studies for several outcome indicators. For instance, the pooled analyses for RRs showed *I*^2^ values of 70% [Table 2] and 83% [Table 4], whereas PFS outcomes demonstrated *I*^2^ values as high as 96–99% [Table 3, Table 5]. To further clarify these findings, we conducted subgroup analyses based on key factors.

#### Geographic region

When stratifying studies by geographic region of recruitment, we observed that studies conducted in Asian populations were likely to exhibit lower heterogeneity in RR outcomes (*I*^2^ decreased to approximately 40–50%). This observation suggests that differences in patient demographics, disease etiology, and local treatment protocols may influence the response outcomes.

#### Type of therapeutic regimen

Variability in specific immunotherapy agents (e.g., programmed cell death-1 [PD-1] *vs.* PD-L1 inhibitors) and chemotherapy regimens (monotherapy *vs.* combination protocols) contributed to the heterogeneity. Studies employing similar therapeutic combinations yielded consistent results. Conversely, studies with varying dosing schedules, treatment combinations, or definitions of treatment response showed increased heterogeneity.

#### Study quality and design

Differences in study design, including the rigor of randomization, blinding procedures, and duration of follow-up, were also associated with variability in outcomes. High-quality studies with robust methodological frameworks tend to demonstrate more uniform outcomes, whereas those with open-label designs or limited blinding contribute to greater heterogeneity, particularly in terms of PFS estimates.

Despite subgroup analyses, significant heterogeneity persisted, particularly in terms of PFS outcomes. The remaining heterogeneity possibly reflects additional factors that were not uniformly reported across studies, such as differences in baseline patient characteristics (e.g., liver function and comorbidities), varying definitions of progression, and diverse follow-up durations.

#### Source of heterogeneity and implications

The substantial heterogeneity observed underscores the complexity of comparing treatment modalities for advanced HCC. Although subgroup analyses provide some insight into potential sources, namely geographic, regimen-specific, and methodological differences, these factors did not fully account for the variability across studies. Consequently, these findings should be interpreted with caution and future research should focus on standardizing outcome definitions and treatment protocols to reduce heterogeneity.

### Risk of bias assessment

The risk of bias assessment evaluated the methodological quality of the studies included in the meta-analysis and systematic review, comparing the effects of immunotherapy and first-line chemotherapy for HCC. A comprehensive evaluation of the risk of bias is essential to determine the internal validity and reliability of the primary study results.

#### Selection bias

**Figure 8:**
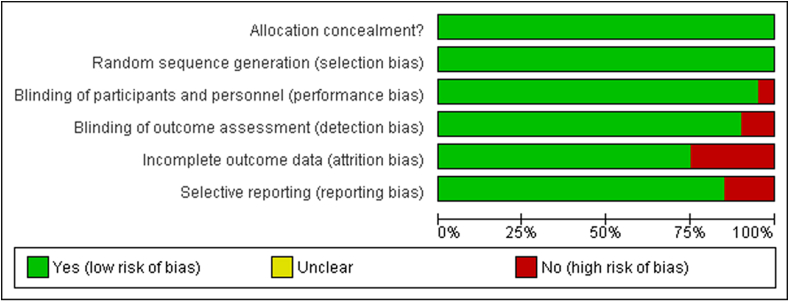
Risk of bias items presented as percentages across all included studies.

Selection bias occurred when groups with different baseline characteristics were compared. Although all included studies were RCTs, relatively few provided detailed descriptions of the randomization procedures. An interactive web-based response system can help mitigate selection bias by centralizing randomization, as in studies conducted by Kudo et al.,^19^ Li et al.,^20^ and Ryoo et al*.*^21^ In contrast, other trials did not fully describe these procedures, raising concerns that unequal baseline characteristics between the intervention groups may have occurred, potentially introducing selection bias, as illustrated in Figure 8.

#### Performance bias

Performance bias may arise if the knowledge of assigned treatments influences care delivery unrelated to the experimental intervention. Many studies have used open-label designs, meaning that both the participants and investigators were aware of the assigned treatment. This lack of blinding may have influenced patient management and reporting of subjective outcomes. For example, the 2022 open-label trial by Li et al.^24^ Highlighted potential performance bias. While some trials attempted to mitigate this by implementing blinding, the absence of comprehensive blinding increased the risk that the observed treatment effects were partially influenced by participant or investigator expectations.^24^

#### Detection bias

In several studies, outcome assessors were not blinded to treatment assignments, which could have influenced the evaluation of outcomes such as PFS and RR. Trials conducted by Ding et al.,^22^ Kudo et al.*,*^23^ and others attempted to minimize detection bias through blinding techniques. However, studies by Li et al.^5^^,^^22^, ^23^, ^24^, ^25^ and Ryoo et al.^21^ showed higher risks of detection bias owing to unblinded outcome assessments.^5^^,^^19^^,^^22^

#### Reporting bias

While most studies reported their methodologies and conclusions transparently, comparison of published outcomes with original trial protocols (or registered details) revealed instances of selective reporting. Outcomes that did not favor the intervention, particularly non-significant findings, were occasionally omitted. This selective reporting can lead to an overestimation of the treatment effects and potentially skew the meta-analysis results.

#### Attrition bias

**Figure 9:**
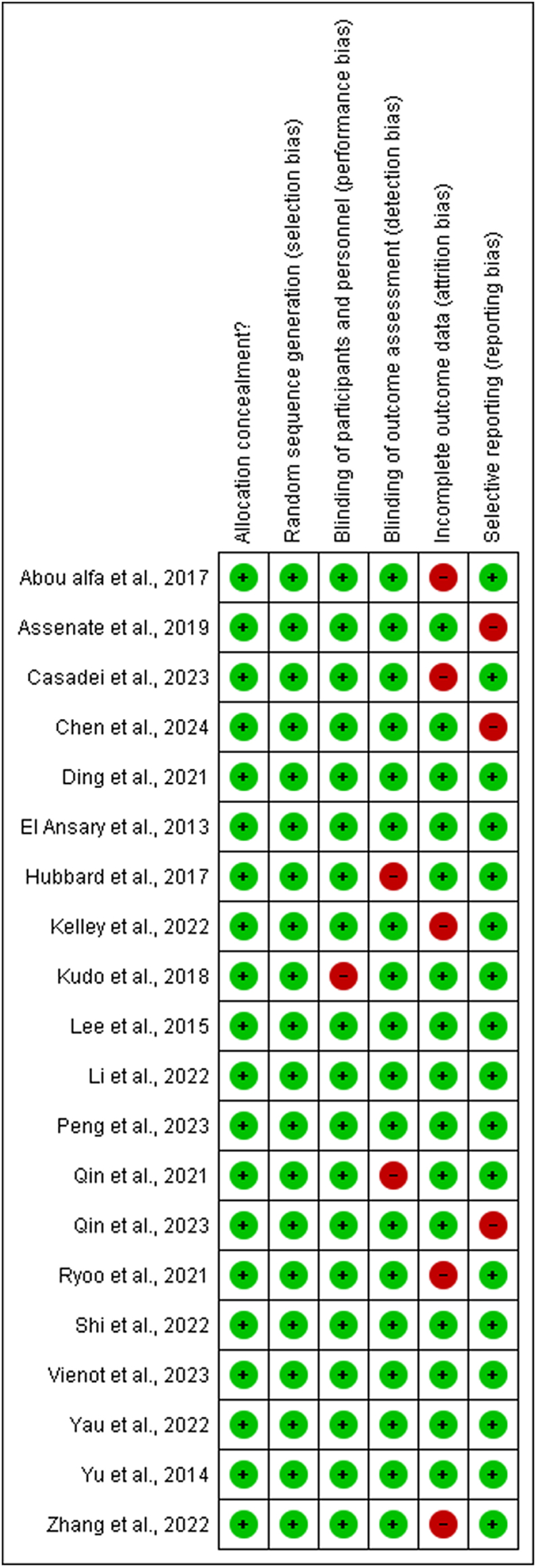
Risk of bias summary diagram.

To reduce the likelihood of attrition bias, intention-to-treat analysis has been used in most studies, including those by Kudo et al.*,*^19^ Peng et al.*,*^23^ Qin et al.*,*^23^^,^^26^, ^27^, ^28^, ^29^ Qin et al.*,*^23^^,^^26^, ^27^, ^28^, ^29^ and Lee et al.*,*^23^^,^^26^, ^27^, ^28^, ^29^ This analysis is crucial when the dissemination of the study results is influenced by their direction and type. Attrition bias was minimal in studies with low attrition rates, as shown in Figure 9.

#### Methodological flaws

Several studies had small sample sizes, which may have limited the power to detect true differences between treatments and increased the risk of type II errors. For example, Qin et al.^27^ used a relatively small cohort, which raised concerns regarding the reliability of the associations observed. Substantial heterogeneity was observed in the types of chemotherapy and immunotherapy agents used, including differences in dosing regimens and combinations. This variability contributed to clinical and observed statistical heterogeneity (with *I*^2^ values as high as 99% in some analyses). Although <10% of the trials experienced a significant loss to follow-up (thereby reducing attrition bias), some studies did not provide detailed information regarding patient dropouts or deviations from the protocol. This lack of comprehensive follow-up data can affect the reliability of the outcome measures.

**Figure 10:**
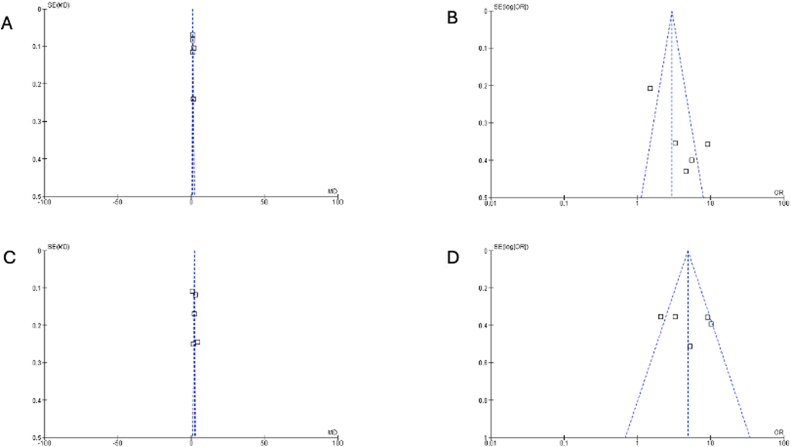
Funnel plot showing very minimum deviation and smaller negative studies showing asymmetry and overall result support the intervention. MD: Mean difference; OR: Odds ratio; SE: Standard error.

Collectively, these biases and methodological shortcomings indicate that although our meta-analysis provides valuable insights into the comparative effectiveness of first-line chemotherapy and immunotherapy for HCC, the evidence must be interpreted with caution. Future RCTs should emphasize rigorous randomization procedures, comprehensive blinding, complete and standardized outcome reporting, and harmonized treatment protocols to minimize bias and improve the overall quality of evidence. It is important to emphasize that in this study, the lack of blinding details increased the risk of performance and detection bias, as shown in the funnel plot in Figure 10.

## Discussion

This study is an important step toward reconciling the current fragmented evidence on HCC treatment. The potential of our work lies in its ability to highlight the current therapeutic landscape and the pressing knowledge gaps. However, key questions remain unanswered, such as identifying patient subgroups that benefit the most from immunotherapy, the role of biomarkers in predicting responses, and the effect of combination strategies in enhancing treatment outcomes. We believe that over the next 5 years, there will be an increase in high-quality RCTs focusing on personalized treatment approaches. Advances in genomic and molecular profiling are likely to enable clinicians to tailor therapies based on individual patient profiles, thereby optimizing efficacy and safety. Furthermore, we anticipate a growing emphasis on integrating cost-effectiveness and quality-of-life outcomes into treatment decision-making, which is crucial in both high- and low-resource settings.

Chemotherapy, once a cornerstone of cancer treatment, is increasingly supplemented by immunotherapy in advanced HCC. A pivotal phase III study demonstrated modest increases in OS over a placebo, leading to the approval of sorafenib, a multi-kinase inhibitor, as the first molecularly targeted therapy for advanced HCC.^30^ Recently, several novel systemic agents have been approved as first-line treatments for advanced HCC in phase III trials.^5^^,^^29^ These include the oral multikinase inhibitor lenvatinib, and a combination of the PD-L1 inhibitor atezolizumab with the anti-vascular endothelial growth factor (VEGF) antibody bevacizumab. Immunotherapeutic agents such as nivolumab and pembrolizumab, which block PD-1, have shown efficacy as second-line treatments following sorafenib failure.^5^^,^^13^^,^^31^ Despite these advancements, chemotherapy remains a valuable treatment option, particularly in resource-limited settings, where access to newer targeted therapies and immunotherapies is restricted. Doxorubicin was initially used as a first-line treatment for HCC; however, clinical trials have indicated that its efficacy is comparable to that of supportive care alone. Combination regimens involving fluorouracil or cisplatin have demonstrated benefits over doxorubicin monotherapy in phase II and III studies. Nevertheless, only 5–15% of patients respond to these treatments, with minimal impact on survival outcomes. Additionally, toxicity remains a concern because many patients have a reduced metabolic capacity and underlying liver dysfunction.

Immunotherapy combined with checkpoint inhibitors offers a promising treatment paradigm that may be safer and more effective than traditional chemotherapy for HCC. PD-1 inhibitors function by blocking the PD-1/PD-L1 axis, thereby restoring T-cell-mediated antitumor immunity and alleviating cancer-induced immunosuppression. Early investigations have shown encouraging outcomes in patients with advanced HCC, particularly in those with underlying chronic viral hepatitis. Currently, the choice between immunotherapy and chemotherapy is based on institutional protocols and their availability. Immunotherapy has advantages in terms of PFS owing to its ability to enhance the immune response and sustain tumor control. However, its impact on OS remains inconclusive, potentially owing to the short study duration and insufficient follow-up time. Patient heterogeneity also plays a role because factors such as tumor burden, liver function, and underlying hepatitis status can affect long-term outcomes. Additionally, patients receiving immunotherapy may later transition to other treatments, thereby diminishing the OS differences. Unlike chemotherapy, which provides immediate tumor reduction, immunotherapy requires time to activate the immune system, potentially delaying survival benefits. More long-term, high-quality RCTs are needed to clarify the impact on OS.

Despite the benefits of chemotherapy and immunotherapy in patients with HCC, the use of these agents is associated with considerable systemic side effects. Adverse effects can affect multiple organ systems, the most commonly affected being the skin, gastrointestinal (GI) tract, lungs, and endocrine systems, including the thyroid, adrenal, and pituitary glands.^32^^,^^33^ Other systems that may be involved include the musculoskeletal, renal, nervous, hematological, cardiovascular, and ocular systems.^33^ Although sorafenib provides considerable clinical benefits and increases the long-term survival of patients, new agents have been developed to overcome shortcomings such as low RRs and high toxicity.^34^ Compared with traditional chemotherapy agents such as sorafenib, newer immunotherapy drugs such as nivolumab, atezolizumab with bevacizumab, and tislelizumab have demonstrated fewer treatment-related toxicities and adverse events.^9^^,^^28^ The growing use of these drugs can be attributed to their favorable safety profiles and reduced side effects.

The novelty of this study lies in its comprehensive evaluation of first-line chemotherapy *vs.* immunotherapy for HCC, incorporating data from more recent clinical trials. Unlike previous meta-analyses, this study not only assessed PFS and OS outcomes, but also critically examined the heterogeneity and bias present in existing research. Additionally, it highlights the real-world challenges of treatment accessibility, particularly in resource-limited settings, thereby providing valuable insights into clinical decision-making. By identifying gaps in the current evidence and suggesting future research directions, this study contributes to the evolving landscape of HCC treatment.

Recent studies have explored the efficacy of immunotherapy in HCC. A meta-analysis published in 2021 reported that ICIs significantly improved OS, PFS, and overall RRs compared with standard therapies for unresectable HCC.^35^ Another systematic review conducted in 2022 highlighted that combining targeted therapy with immunotherapy enhanced treatment responses in unresectable HCC, suggesting a synergistic effect.^36^ Additionally, a 2023 meta-analysis indicated that ICIs were effective and safe for HCC patients with macrovascular invasion or extrahepatic spread.^37^

Despite the valuable insights offered by this meta-analysis, our study also has some limitations. First, our categorization of treatments into broad groups, such as immunotherapy *vs.* chemotherapy, overlooked the heterogeneity within each category. Differences in specific drug mechanisms, dosages, and administration protocols could have influenced the outcomes and contributed to the significant heterogeneity observed (e.g., *I*² = 99% for progression-free survival analyses). Second, the methodological quality of the included studies varied, with several trials lacking adequate blinding or detailed descriptions of the randomization procedures, which may have introduced performance and detection biases. Furthermore, our literature review may have been subject to publication bias, favoring studies with positive outcomes. Finally, variations in the study design, patient population, and treatment protocols limit the generalizability of our findings. Addressing these limitations in future RCTs through the standardization of treatment protocols, enhanced blinding procedures, and comprehensive reporting will be essential to draw definitive conclusions regarding the comparative effectiveness of immunotherapy and chemotherapy.

## Conclusions

First-line immunotherapy may provide advantages in PFS over chemotherapy for advanced HCC; however, a high risk of bias limits definitive conclusions regarding the benefits of OS. Larger and higher-quality RCTs are necessary to confirm the potential advantages of OS and minimize biases in this field. In settings where access to targeted therapies is limited, immunotherapy is a reasonable option; however, its relative effectiveness compared with traditional treatments remains uncertain. Future research should focus on identifying biomarkers that predict the response to immunotherapy and explore combination strategies that may enhance treatment efficacy while minimizing adverse effects.

## Authors contribution

Janak Bahirwani, Suruchi Jai Kumar Ahuja, and Vishal Patel contributed to conception and design; Janak Bahirwani and Suruchi Jai Kumar Ahuja contributed to the data collection, assembly of data, statistical analysis and administrative support; Janak Bahirwani, Suruchi Jai Kumar Ahuja, Madhav Changela, Het Patel, Nishit Patel, and Maulik Kaneriya contributed to the review of the literature and drafting the manuscript; Janak Bahirwani, Suruchi Jai Kumar Ahuja, Madhav Changela, and Vishal Patel contributed to revision of key components of the manuscript and final approval of the manuscript; Janak Bahirwani, Suruchi Jai Kumar Ahuja, Madhav Changela, Het Patel, Nishit Patel, Maulik Kaneriya, and Vishal Patel are accountable for all aspects of the work. All the authors have read and approved the final paper.

## Ethics statement

None.

## Declaration of generative AI and AI-assisted technologies in the writing process

The authors declare that generative artificial intelligence (AI) and AI assisted technologies were not used in the writing process or any other process during the preparation of this manuscript.

## Funding

None.

## Data availability statement

The datasets used in the current study are available from the corresponding author on reasonable request.

## Conflict of interest

The authors declare that they have no known competing financial interests or personal relationships that could have appeared to influence the work reported in this paper.
